# CRISPR/Cas9-mediated CH2 deletion of Fel d1 triggers transcriptomic reprogramming and disease-associated pathways in feline cells

**DOI:** 10.3389/fcell.2025.1716808

**Published:** 2025-11-21

**Authors:** Lin-Yi Qu, Fu-Shi Quan, Shu-Ming Shi, Zhi-Chao Chi, Jian Chen, Yong-Xun Jin, Ming-Jun Zhang, Il-Keun Kong, Xian-Feng Yu

**Affiliations:** 1 College of Animal Sciences, Jilin University, Changchun, Jilin, China; 2 Department of Animal Science, Division of Applied Life Science (BK21 Four Program), Gyeongsang National University, Jinju, Gyeongnam, Republic of Korea

**Keywords:** Fel d1, CRISPR/Cas9, transcriptome sequencing, hypertrophic cardiomyopathy, rheumatoid arthritis, CH2

## Abstract

Fel d1, the major cat allergen responsible for over 90% of human IgE-mediated allergies, has an incompletely defined physiological role. To explore its function and assess the feasibility of producing hypoallergenic cats, we knocked out the CH2 domain of Fel d1 using CRISPR/Cas9 in feline skin cells. An optimized sgRNA introduced a frameshift mutation, with knockout efficiency validated by sequencing, qRT-PCR, and Western blot. Transcriptomic alterations were profiled by RNA-seq, and functional consequences were investigated via GO, KEGG, and GSEA analyses. Key findings were confirmed by qPCR, and phenotypes were assessed using CCK-8, EdU, and flow cytometry. The approach successfully generated a three-base insertion, resulting in near-complete loss of CH2 mRNA and Fel d1 protein. RNA-seq identified 3,469 differentially expressed genes (DEGs), with significant enrichment in pathways for hypertrophic cardiomyopathy (HCM) and rheumatoid arthritis (RA). Key genes in these pathways (e.g., *TGFB2*, *MYBPC3*, *MMP3*, and *TLR4*) were upregulated, and CH2 deletion impaired proliferation while increasing apoptosis. We conclude that CH2 deletion, while effectively abolishing the major allergen, triggers unintended transcriptomic reprogramming linked to pathological states. This underscores the necessity of comprehensive safety profiling, including transcriptomics, prior to applying gene-edited cells in SCNT-based development of hypoallergenic cats.

## Highlights

- CRISPR/Cas9 knockout of Fel d1 CH2 domain achieved in primary feline skin cells
- Transcriptome profiling revealed enrichment of RA and HCM-related signaling pathways
- CH2 deletion induced aberrant expression of genes involved in fibrosis and inflammation

## 1 Introduction

The prevalence of pet ownership has risen globally, with domestic cats (*Felis catus*) being one of the most popular companion animals (Bredemeyer et al., 2021). Cat allergy is increasingly prevalent worldwide, with approximately 15% of the population exhibiting allergic symptoms—typically rhinoconjunctivitis and asthma—especially in urban areas (Chan and Leung, 2018). Fel d1, the major cat allergen secreted by sebaceous and salivary glands, acts as the principal sensitizing agent in allergic reactions (Schoos et al., 2021; Kiewiet et al., 2023). This protein is highly stable, persisting in indoor environments for months even after cat removal, and readily binds to airborne particles to amplify allergic responses (Maya-Manzano et al., 2022). Structurally, Fel d1 is a 35 kD tetramer composed of two heterodimers, each consisting of two chains: chain 1 (70 aa, 8 kD) encoded by the CH1 gene, and chain 2 (92 aa, 10 kD) encoded by the CH2 gene (Griffith et al., 1992). Multiple sequence comparisons have shown that the Fel d1 gene sequence is not conserved, and its relatively low sequence identity (95.9% and 93.5% for the CH1 and CH2 genes, respectively, in 24 exotic cats) -a pattern comparable to the genome-wide average of non-coding regions (Cho et al., 2013). This lack of evolutionary conservation implies that the Fel d1 gene may not be essential for cats and suggests that the CH1 or CH2 genes may be suitable targets for gene knockout. Further supporting this approach, the Fel d1 gene is expressed exclusively in felines (Brackett et al., 2022). Traditional allergen avoidance and treatments (e.g., antihistamines, allergen-specific immunotherapy (AIT)) offer only partial relief and fail to eliminate Fel d1 at its source, often requiring long-term use with inconsistent outcomes (Trifonova et al., 2025). More recently, strategies such as immunization and antibody neutralization have been developed to reduce environmental Fel d1 exposure (Satyaraj et al., 2019). However, as Fel d1 is the major allergen in cats (Ukleja-Sokolowska et al., 2016), a truly fundamental solution requires preventing its production entirely. In this regard, gene-level deletion represents the most definitive approach to abolish Fel d1 expression at its genetic source.

The CRISPR/Cas9 system directs the Cas9 nuclease to a specific DNA sequence via a single-guide RNA (sgRNA) (Ma et al., 2014). It recognises the PAM sequence and induces double-strand breaks (DSBs) (Jinek et al., 2012), which initiates endogenous cellular repair mechanisms such as nonhomologous end joining (NHEJ), microhomology mediated end joining (MMEJ), and homologous recombination (HR) (Salsman and Dellaire, 2017). Currently, Crispr/Cas9 gene editing technology has been widely used and combined with Somatic cell nuclear transfer (SCNT) to generate gene-edited animals in pigs (Eun et al., 2022), sheep (Zhang Y. et al., 2019), cows (Suva et al., 2025), and so on. Thus, CRISPR/Cas9-mediated Fel d1 gene editing represents one of the most fundamental and promising strategies for addressing cat allergy. Recent innovations aim to reduce the major cat allergen Fel d1 at its source, building on early successes in using CRISPR/Cas9 technology in cats (Brackett et al., 2022). As a heterodimer, the functional integrity of Fel d1 is contingent upon both its constituent chains. This makes the CH2 gene, which encodes one of these essential chains, a critical target for ablation. However, the specific physiological role of the CH2 chain, beyond its contribution to allergen structure, remains entirely unknown. This gap in knowledge presents a significant hurdle, as unintended consequences of its deletion on cellular homeostasis cannot be predicted.

Domestic cats are susceptible to a spectrum of chronic diseases that significantly impact their welfare and longevity. Among these, HCM and RA represent two prevalent conditions. HCM, the most common feline myocardial disorder, is characterized by primary inappropriate left ventricular hypertrophy, leading to diastolic dysfunction, reduced cardiac compliance, and impaired ventricular filling (Kittleson and Cote, 2021a). This pathology affects approximately 15% of the general cat population (Kittleson and Cote, 2021b), with higher prevalence in middle-aged males and specific breeds such as Maine Coon and Ragdoll, due to identified genetic mutations (e.g., MyBPC gene) (Messer et al., 2017). Rheumatoid arthritis in cats is an immune-mediated, erosive polyarthropathy driven by synovial inflammation and progressive cartilage destruction (Lemetayer and Taylor, 2014). Given the uncertainty surrounding the physiological function of Fel d1 and the potential risks posed by gene editing, transcriptomic profiling provides an essential tool to uncover unintended molecular consequences. High-throughput RNA sequencing enables unbiased detection of gene expression alterations and can reveal hidden perturbations in pathways related to immunity (Gill and Dhillon, 2022), fibrosis, or development—key concerns when evaluating the safety of gene-edited donor cells for SCNT applications. This study employed CRISPR/Cas9-mediated gene editing to ablate the CH2 gene in cat skin cells, thereby abolishing expression of the major allergen Fel d1. Concomitantly, RNA sequencing (RNA-seq) and Gene Set Enrichment Analysis (GSEA) were integrated to systematically delineate transcriptomic alterations and pathway-level perturbations resulting from CH2 knockout.

## 2 Materials and methods

### 2.1 Primary cell isolation and culture

All experiments were performed with at least three biological replicates. Full-thickness skin biopsies (≥4 mm^2^) were collected during surgical procedures. Immediately place in sterile PBS supplemented with 2× antibiotics (200 U/mL penicillin, 200 μg/mL streptomycin) and transport on ice ≤2 h to the laboratory. Upon receipt of the sample, it was sequentially disinfected with 75% ethanol (30 s), followed by a thorough rinse with PBS to remove residue. After removal of subcutaneous fat with iris scissors, the tissue was cut into pieces less than 0.5 cm. Afterwards, the cells were digested with collagenase IV (1.5 mg/mL) in an incubator (37 °C, 5% CO_2_) for 2–4 h. The cell suspension was filtered, centrifuged (200 × g, 5 min), and resuspended in culture medium (DMEM; Gibco™, United States) with 10% heat-inactivated fetal bovine serum (FBS), 100 U/mL penicillin, and 100 μg/mL streptomycin. Cells were seeded into 100 mm Cell Culture Dishes and maintained in culture at 37 °C, 5% CO_2_ with medium renewal every 48 h.

### 2.2 sgRNA design and package delivery

SgRNAs targeting the cat CH2 gene (NCBI RefSeq: NC_058382.1) were computationally designed using CRISPR RGEN Tools (http://www.rgenome.net/). The oligonucleotide sequences are detailed in Table 1. The sgRNA oligonucleotide duplexes were annealed and ligated into the BbsI-linearized PX459 vector (Addgene #62988) with the pSpCas9 protein sequence, and then transform into competent DH5α cells (TIANGEN Biotech, CB101,China) under ampicillin selection. Transfection into cells was performed via Liposome 3,000 (Thermofisher, L3000001).

**TABLE 1:** sgRNA detailed sequence and PAM regions in the following table.

sgRNA	Sequence (5′-3′)	PAM (5′-3′)
sgRNA-1	F:CCAAGCGCTGGGCGTCAAGA R:TCTTGACGCCCAGCGCTTGG	TGG
sgRNA-2	F:GACTAGTCCATCCAAGACCC R:GGGTCTTGGATGGACTAGTC	TGG
sgRNA-3	F:AGCCTGTTCTACCACACGTG R:CACGTGTGGTAGAACAGGCT	GGG

### 2.3 DNA extraction and PCR

Genomic DNA was isolated from wild-type and CH2-knockout (KO) cell lines using the TIANGEN Genomic DNA Kit (DP304, China) following the manufacturer’s protocol, with DNA concentration quantified spectrophotometrically (NanoDrop™ 2000). Flanking regions of the sgRNA target sites were amplified by PCR (F: TGAGCAGAGCATTCTAGCAG, R: TCTATGCCACACCGATATTAGT), using locus-specific primers designed based on NC_058382.1 coordinates. The PCR program is shown in Table 2.

**TABLE 2:** PCR program in the following table.

Cycle step	Temp	Time	Cycle number
Initial denaturation	95 °C	2 min	1
Denaturation	95 °C	30 s	35
Annealing	58 °C	30 s	35
Extension	72 °C	1 min	35
Final extension	72 °C	10 min	1

### 2.4 T7EI cleavage assay

The T7 endonuclease I (T7EI) assay was used to detect CRISPR/Cas9-induced indel mutations. Genomic DNA from CRISPR-edited polyclonal populations was isolated using the TIANGEN Genomic DNA Kit (DP304, China). Target loci were amplified with LA Taq® DNA Polymerase (TaKaRa Bio Inc., Japan) under standard cycling conditions (as shown in Table 2). Purified amplicons (QIAquick PCR Purification Kit, Qiagen) were subjected to T7EI digestion (New England Biolabs, United States) at 37 °C for 15 min. Cleavage products were resolved on 1% agarose gels stained with GelRed™ (Biotium) and quantified using NIH ImageJ software 1.52a (National Institutes of Health, Bethesda, MD, United States) and the editing efficiency was calculated by using the formula i.e., efficiency = [(sum of cleaved band intensities)/sum of cleaved and parental band intensities)] × 100.

### 2.5 Single cell isolation

To generate monoclonal cell lines, single-cell isolation was performed after puromycin selection. Cells were serially diluted and seeded into 96-well plates to achieve approximately 0.5–1 cell per well, ensuring clonality. After 48 h of transfection and 3 days of screening by 5 μg/mL puromycin, cells were digested with trypsin, centrifuged, and the supernatant was discarded and mixed; the number of viable cells was counted using a cell counting plate; the cells were diluted according to containing 500–600 cells per 10 mL, and after dilution, 2 rows were inoculated by adding 0.1 mL of cell suspension into each well of a 96-well plate; the remaining cell suspension was inoculated by continuing to dilute the cells with the culture medium at a multiplicity ratio, then inoculated with 2 rows, and so on, until each well contained half or 1 cell. Plates were then microscopically examined within 24 h to identify and mark wells containing exactly one adherent cell for clonal expansion. A total of thirty-five 96-well plates were seeded to ensure sufficient monoclonal recovery. Single-cell-derived clones at passage 3 (P3) were used for subsequent validation experiments.

### 2.6 Quantitative real-time PCR (qRT-PCR)

Total mRNA was isolated from control and CH2-KO cells by using mRNA isolation kit (QIAGEN, Germany, 80,284), and gene expression was assessed using a Thermo Fisher Scientific 6/7/12K Flex RT-PCR system, with the PCR procedures detailed in Table 3. GAPDH expression was used as an internal control, as detailed in Table 4.

**TABLE 3:** qRT-PCR program in the following table.

Cycle step	Temp	Time	Cycle Number
Initial denaturation	95 °C	5 min	1
Denaturation	95 °C	20 s	40
Annealing	60 °C	20 s	40
Extension	72 °C	20 s	40
Final extension	72 °C	10 min	1

**TABLE 4:** The tested genes, primer sequences, PCR product sizes, and accession numbers for qRT-PCR experiments are provided in the following table.

Gene name	Sequence	Amplicon size (bp)	Accession number
*CH2*	F: GGCGTCAAGATGGCGGAAACR: ATGGCTGTTCTCTCTGGTTCAG	128	NM_001048154.1
*TGFB2*	F:CTGTGGGTACCTTGATGCCAR:CTCCATCGCTGAGACGTCAA	82	XM_003999507.6
*TGFB3*	F:AGCATTCACTGTCCGTGTCAR:CCTCGCTGTCCACACCTTT	100	XM_003987851.4
*ITGA2*	F:AGAACCGAATGGGAGACGTGR:ATGCTTGTGGCAGTTTGCAG	88	XM_045037280.1
*ITGB3*	F:GGTAGAGGAGCCAGAGTGTCR:CAGAGTAGCAAGGCCGATGA	94	XM_003997035.6
*MYBPC3*	F:CGGGGAAGAGCCAGTCTCAGR:TGTCTCGGCCTCGAACACAG	89	XM_019812397.2
*MMP1*	F:CCTTTGTGTGGGGAGATCACTR:TGGAAAGCATGAGCGAGGTT	73	XM_003992316.4
*MMP3*	F:TTTGTCTGCCAGTCTGCTCTCTR:CTCCAGGTATTGCTGGACAA	89	XM_003992306.2
*TLR4*	F:GGACCCTTGTGTGGAGGTGGR:GGGATGTTGTCGGGGATTTTG	81	NM_001009223.1
*GAPDH*	F: GTCGGTGTGAACGGATTTGGR: GCCGTGGGTGGAATCATACT	147	NM_001009307.1

### 2.7 Western blot analysis

Cells are cultured and harvested using Lysis Buffer containing a mixture of protease and phosphatase inhibitors. Electrophoresis was performed using SDS-PAGE precast gels. Proteins were then transferred to PVDF membranes, which were subsequently enclosed in TBST (20 mM Tris-HCl [pH 7.5], 150 mM NaCl, and 0.05% Tween-20) containing 5% skimmed milk for 1 h. We then applied diluted primary antibodies: Fel d1 (1:500, FELD1-112AP, Thermofisher) to the membrane, incubating them overnight at 4 °C. The membranes were subsequently washed five times with TBST and incubated with a horseradish peroxidase-conjugated secondary antibody (1:5,000; Proteintech, SA00001–1) for 1 h. After incubation, each membrane underwent five TBST washes (10 min each time) and was developed using enhanced chemiluminescence solution (Tanon, 180–5,001) at room temperature for 5 s. Target protein expression was quantified with β-actin (1:1,000, 66009-1-Ig, Proteintech) as the reference control.

### 2.8 Cell Viability and proliferation assays

Cell viability was determined using the Cell Counting Kit-8 (CCK-8; Beyotime, China, Cat# C0038). Control and CH2-KO cells were seeded in 96-well plates at a density of 3 × 10^3^ cells/well and cultured for 12, 24, 48, and 72 h. At each time point, 10 μL of CCK-8 reagent was added to each well, followed by incubation at 37 °C with 5% CO_2_ for 2 h. The absorbance was then measured at 450 nm using a microplate reader. Cell proliferation was directly assessed using the BeyoClick™ EdU Cell Proliferation Kit with Alexa Fluor 594 (Beyotime, China, C0071S) according to the manufacturer’s protocol. Briefly, cells were seeded in 24-well plates and incubated with 10 μM EdU for 2 h at 37 °C. After fixation and permeabilization, the Click-iT reaction cocktail was applied to detect the incorporated EdU. Cell nuclei were counterstained with DAPI. Images were captured using a fluorescence microscope, and the percentage of EdU-positive cells was quantified from at least three random fields per well using ImageJ software.

### 2.9 Apoptosis analysis by flow cytometry

Cells were inoculated into 6-well plates (5 × 10^5^ cells/well) and cultured for 24 h. After trypsinization of the cells with ethylenediaminetetraacetic acid-free trypsin (Gibco, United States, Cat# 25200056), the cells were stained with Annexin V-FITC Apoptosis Detection Kit (Beyotime, China, Cat# C1383M) according to the manufacturer’s protocol. Briefly, cells were resuspended in 100 μL of 1× binding buffer containing 5 μL of Annexin V-FITC and 5 μL of propidium iodide (PI) and then incubated for 15 min at 25 °C in the dark. Immediately prior to analysis, 400 μL of binding buffer was added to each sample. Data acquisition was performed on a BD FACSCanto II flow cytometer with 488 nm excitation and 10,000 events per sample. FlowJo v10.8 software was used to analyze surviving cells (Annexin V-/PI-), early apoptotic cells (Annexin V^+^/PI-), late apoptotic cells (Annexin V^+^/PI^+^), and necrotic cells (Annexin V-/PI^+^) were quantitatively analyzed. Repeat technology measurements of three biological repeat sequences were analyzed. Three independent biological replicates were analyzed.

### 2.10 Transcriptome data analysis

Total RNA was extracted using Trizol reagent (Invitrogen), with concentration, purity, and integrity assessed using a NanoDrop spectrophotometer (Thermo Scientific). Using 3 μg RNA as input, poly(A)+ mRNA was isolated with oligo (dT)-attached magnetic beads. mRNA fragmentation was performed in Illumina proprietary buffer containing divalent cations under elevated temperature. First-strand cDNA synthesis utilized random oligonucleotide primers and SuperScript II reverse transcriptase, followed by second-strand synthesis with DNA Polymerase I and RNase H. cDNA ends were blunted via exonuclease/polymerase activity and enzymes were removed. Following 3′end adenylation, Illumina PE adapters were ligated. Libraries were size-selected (400–500 bp) using AMPure XP beads (Beckman Coulter). Adapter-ligated fragments were PCR-amplified for 15 cycles with Illumina PCR Primer Cocktail. PCR products were purified (AMPure XP) and quantified using the Agilent High Sensitivity DNA kit on a Bioanalyzer 2,100 system (Agilent). Final libraries were sequenced on Illumina’s NovaSeq 6,000 platform at Shanghai Personal Biotechnology Co., Ltd. Three biological replicates per group (Control and CH2-KO) were sequenced.

### 2.11 Differential genes and enrichmentexpression analysis

Differential gene expression analysis was performed using the DESeq2 R package (v1.38.3). Genes with an adjusted P-value <0.05 and an absolute log_2_ fold change >1 were defined as differentially expressed. The pheatmap R package (v1.0.12) was used for bidirectional hierarchical clustering of all genes, using Euclidean distance and complete linkage methods. Gene Ontology (GO) enrichment analysis was performed on the lists of differentially expressed genes using the top GO (v2.50.0) and the hypergeometric test with a significance threshold of P-value <0.05. Kyoto Encyclopedia of Genes and Genomes (KEGG) pathway enrichment analysis was executed using the clusterProfiler R package (v4.6.0), with a significance threshold of P-value <0.05. Gene Set Enrichment Analysis (GSEA) was performed on the entire ranked gene list using GSEA software (v4.1.0).

### 2.12 Statistical analysis

SPSS software version 11.0 (IBM, United States) for data analysis. Each experiment was conducted at least thrice. For comparisons between two groups, data were analyzed using two-tailed unpaired t-test. For multiple group comparisons, one-way ANOVA with Tukey’s post-hoc test was used. Data comparisons between groups were done using t-test. Results are presented with mean ± standard deviation, with significant differences indicated by *(P < 0.05), **(P < 0.01), *** (P < 0.001) and **** (P < 0.0001).

## 3 Results

### 3.1 Screening and optimization of sgRNAs targeting the feline CH2 gene

**FIGURE 1:**
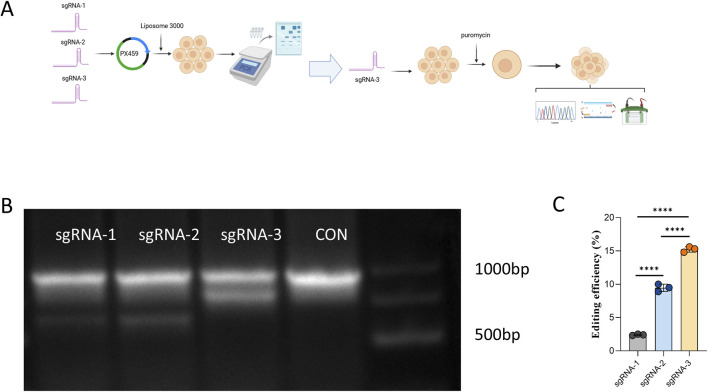
Screening and Optimization of sgRNAs Targeting the Feline CH2 Gene. **(A)** Experimental workflow showing the design of three sgRNAs targeting the feline CH2 gene, transfection into cells, and subsequent selection and screening. **(B)** Electrophoresis of T7E1-digested PCR products showing cleavage patterns for each sgRNA. **(C)** Quantitative analysis of genome editing efficiency for each sgRNA. Data were obtained from three independent experiments. Significant differences are indicated by **** (P < 0.0001).

As illustrated in Figure 1A, three candidate sgRNAs (sgRNA-1, sgRNA-2, and sgRNA-3) targeting distinct functional domains of the feline CH2 gene (*Felis catus*; NCBI RefSeq: NC_058382.1) were designed using the CRISPR RGEN online tool. The sequences of these sgRNAs are listed in Table 1. Each sgRNA construct was transfected into cells using Lipofectamine™ 3,000, followed by puromycin selection to enrich for positively transfected cells. Genomic DNA was extracted from the pooled populations, and site-specific PCR was performed flanking each sgRNA target site. PCR products were subjected to T7E1 digestion and separated via 1% agarose gel electrophoresis to assess editing efficiency (Figure 1B). The cleavage products indicated the presence of a heterogeneous mixture of indels in the polyclonal population. Among the three sgRNAs, sgRNA-3 exhibited the highest gene editing efficiency (Figure 1C), as determined by T7E1 cleavage assay and quantified using ImageJ, and was therefore selected as the optimal candidate for subsequent single-cell cloning.

### 3.2 Generation of CH2-Knockout monoclonal cells

**FIGURE 2:**
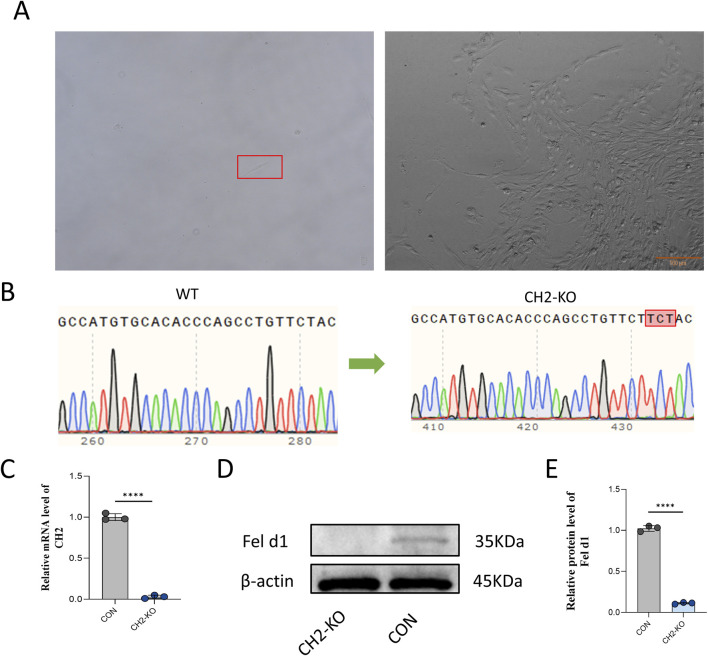
Generation of CH2-Knockout Monoclonal Cells. **(A)** Monoclonal selection of cells after puromycin screening. Scale bar: 100 µm. **(B)** Sanger sequencing showing a three-base insertion mutation at the target site in the CH2-KO clone. **(C)** qRT-PCR results for *CH2* mRNA expression in control and KO cells. **(D)** Western blot analysis of Fel d1 protein expression in control and KO cells. **(E)** Quantification of Fel d1 protein expression levels. Data were obtained from three independent experiments. Data are presented as mean ± standard deviation, and P values were calculated by two-tailed unpaired t-test. Significant differences are indicated by **** (P < 0.0001).

After 48 h of sgRNA-3 transfection into cells, puromycin selection (5 μg/mL, 5 days) was applied, followed by limited dilution in 96-well plates to isolate monoclonal cell lines (Figure 2A). PCR amplification and Sanger sequencing confirmed a three-base insertion mutation at the target site in the monoclonal clone (Figure 2B). This specific 3-bp insertion was identified within a critical coding region of the CH2 gene. The mutation is predicted to cause a frameshift that introduces a premature termination codon (PTC) downstream, leading to the production of a truncated and non-functional CH2 protein, or alternatively, triggering nonsense-mediated decay (NMD) of the mutant mRNA. The knockout efficiency was validated at both mRNA and protein levels by qRT-PCR and Western blotting (Figures 2C–E), showing near-complete loss of CH2 transcript and Fel d1 protein expression. The absence of Fel d1 protein is consistent with the predicted loss-of-function (LOF) outcome of the frameshift mutation. These results confirmed the successful establishment of a CH2 gene knockout cell line via CRISPR/Cas9.

### 3.3 Transcriptomic profiling reveals global reprogramming induced by CH2 knockout

**FIGURE 3:**
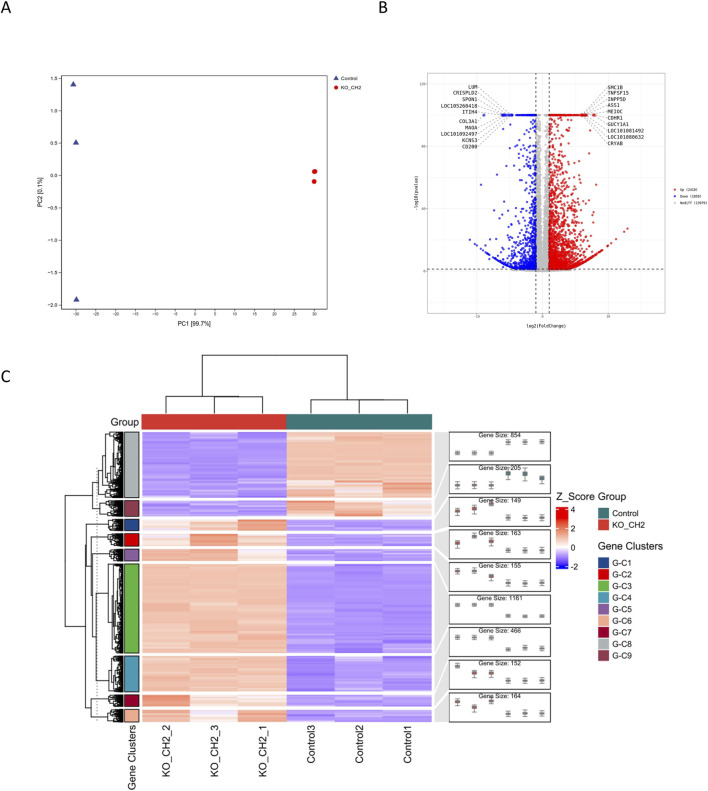
Transcriptomic Profiling Reveals Global Reprogramming Induced by CH2 Knockout. **(A)** Principal component analysis (PCA) showing distinct transcriptomic separation between control and CH2-KO groups. **(B)** Volcano plot showing DEGs in CH2-KO versus control group. **(C)** Hierarchical clustering heatmap showing 9 gene expression clusters among DEGs.

To explore the potential biological function of Fel d1, a secretory protein encoded by CH2 and CH1, we performed transcriptome analysis in cells. Given that Fel d1 may be involved in nuclear suppression and cellular phenotype establishment, RNA-seq was conducted to elucidate the molecular consequences of CH2 knockout. Principal component analysis (PCA) revealed a clear separation between the CH2-KO and control groups (Figure 3A), indicating substantial transcriptomic differences. Volcano plot analysis identified 2,410 significantly upregulated and 1,059 significantly downregulated genes in CH2-KO cells compared to controls (Figure 3B), we list the 40 genes with the greatest degree of variation in Table 5. Hierarchical clustering of DEGs revealed nine gene co-expression clusters (Figure 3C), each representing genes with similar expression profiles across samples, suggesting coordinated transcriptional reprogramming. These modules indicate functional remodeling, including activation of chromatin regulation pathways and suppression of extracellular matrix biosynthesis. GO and KEGG pathway enrichment were subsequently conducted to investigate biological processes and disease-related pathways, suggesting widespread reprogramming induced by CH2 deletion.

**TABLE 5:** The most significantly expressed 40 genes of DEGs in the control and KO-CH2 groups.

Gene symbol	Chromosome	Gene name	log2FoldChange
*GNA15*	NC_058369.1	G protein subunit alpha 15	−11.06
*NRN1*	NC_058372.1	Neuritin 1	−10.3
*SHOX2*	NC_058376.1	Short stature homeobox 2	−9.985
*PDGFD*	NC_058377.1	Platelet derived growth factor D	−9.384
*FGF10*	NC_058368.1	Fibroblast growth factor 10	−9.354
*NCAM1*	NC_058377.1	Neural cell adhesion molecule 1	−9.047
*COL3A1*	NC_058375.1	Collagen type III alpha 1 chain	−8.908
*IGFBP3*	NC_058369.1	Insulin like growth factor binding protein 3	−8.677
*MKX*	NC_058374.1	Mohawk homeobox	−8.663
*TMEM132C*	NC_058379.1	Transmembrane protein 132C	−8.476
*GABRA2*	NC_058371.1	Gamma-aminobutyric acid type A receptor alpha2 subunit	−8.38
*HOXB3*	NC_058381.1	Homeobox B3	−8.34
*KIT*	NC_058371.1	KIT proto-oncogene, receptor tyrosine kinase	−8.291
*LPL*	NC_058371.1	Lipoprotein lipase	−8.07
*C2CD6*	NC_058375.1	C2 calcium dependent domain containing 6	−7.545
*LAMA2*	NC_058372.1	Laminin subunit alpha 2	−7.356
*PREX2*	NC_058385.1	Phosphatidylinositol-3,4,5-trisphosphate dependent Rac exchange factor 2	−7.349
*LRATD1*	NC_058370.1	LRAT domain containing 1	−7.195
*EBF2*	NC_058371.1	EBF transcription factor 2	−7.172
*SHOX*	NC_058386.1	Short stature homeobox	−7.164
*HOXB2*	NC_058381.1	Homeobox B2	−7.077
*FGF13*	NC_058386.1	Fibroblast growth factor 13	−7.041
*SLITRK6*	NC_058368.1	SLIT and NTRK like family member 6	−6.931
*PLA2G7*	NC_058372.1	Phospholipase A2 group VII	−6.92
*ZNF215*	NC_058377.1	Zinc finger protein 215	−6.781
*HOXD4*	NC_058375.1	Homeobox D4	−6.694
*HOXB4*	NC_058381.1	Homeobox B4	−6.648
*PCSK5*	NC_058380.1	Proprotein convertase subtilisin/kexin type 5	−6.539
*ALX4*	NC_058377.1	ALX homeobox 4	−6.379
*LRRC17*	NC_058369.1	Leucine rich repeat containing 17	−6.314
*FREM1*	NC_058380.1	FRAS1 related extracellular matrix protein 1	−6.31
*PLCB4*	NC_058370.1	Phospholipase C beta 4	−6.212
*EPHA4*	NC_058375.1	EPH receptor A4	−6.161
*ITIH4*	NC_058369.1	Inter-alpha-trypsin inhibitor heavy chain 4	−6.138
*CCN5*	NC_058370.1	Cellular communication network factor 5	−6.117
*FUT1*	NC_058382.1	Fucosyltransferase 1	−6.102
*HDX*	NC_058386.1	Highly divergent homeobox	−6.095
*PIFO*	NC_058375.1	Primary cilia formation	−6.091
*THSD7A*	NC_058369.1	Hrombospondin type 1 domain containing 7A	−5.997
*UBASH3B*	NC_058377.1	Ubiquitin associated and SH3 domain containing B	−5.986

### 3.4 CH2 deficiency alters key signaling pathways and cell viability

**FIGURE 4:**
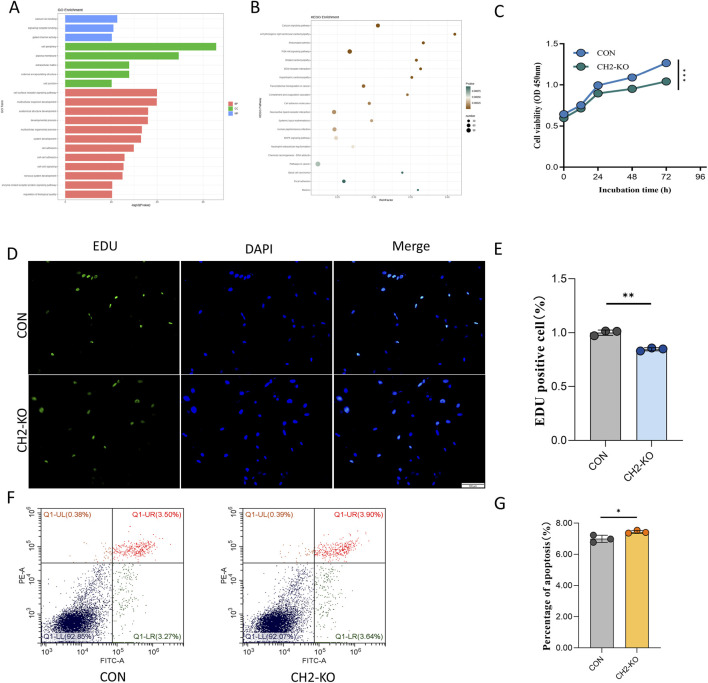
CH2 Deficiency Alters Key Signaling Pathways and Cell Viability. **(A)** GO enrichment analysis showed that DEGs after CH2 knockout were mainly enriched in molecular functions such as calcium ion binding, gated channel activity, and receptor ligand activity; cellular components including plasma membrane and extracellular regions; and biological processes related to signal transduction and developmental regulation. **(B)** KEGG pathway enrichment analysis reveals significant enrichment in calcium signaling pathway, arrhythmogenic right ventricular cardiomyopathy (ARVC), and rheumatoid arthritis, suggesting potential links between CH2 deletion and cardiovascular and autoimmune dysregulation. **(C)** Time-course CCK-8 proliferation assay in control and CH2-KO cells. While proliferation remains comparable at 12 h and 24 h, a significant reduction in growth is observed at 48 h and 72 h in CH2-KO cells, indicating impaired proliferation over time. **(D)** Representative images of EdU staining (green: EdU; blue: DAPI) in control and CH2-KO cells. Scale bar: 100 μm. **(E)** Quantification of EdU-positive cells show a significant decrease in the proliferation rate of CH2-KO cells compared to controls. **(F)** Flow cytometric analysis of apoptosis rate in control and CH2-KO cells. Increased PI-positive populations indicate enhanced apoptosis in knockout cells. **(G)** Quantification of apoptotic rates based on flow cytometry, confirming increased cell death following CH2 knockout. Significant differences are indicated by * (P < 0.05), ** (P < 0.01) and *** (P < 0.001).

To further investigate the impact of CH2 knockout on cellular function, RNA-seq analysis was conducted in CH2-KO cells. DEGs were subjected to GO and KEGG enrichment analyses. GO annotation revealed significant enrichment of DEGs in molecular functions such as “calcium ion binding (GO:0005509),” “signaling receptor binding (GO:0005102),” and “gated channel activity (GO:0022836),” indicating a potential role in ion homeostasis and transmembrane signal transduction. In terms of cellular components, DEGs were predominantly enriched in “membrane region,” “extracellular region,” and “cell–cell junction,” highlighting their involvement in intercellular communication and microenvironmental regulation. Biological process enrichment emphasized transcriptional regulation of “cell surface receptor signaling pathways,” “organ morphogenesis,” and “multicellular organismal development,” suggesting functional reprogramming of morphogenetic and signaling pathways (Figure 4A).

KEGG pathway analysis further confirmed that DEGs were significantly enriched in multiple signaling and disease-associated pathways. Among these, the “calcium signaling pathway” showed the highest enrichment score, consistent with GO findings. Notably, several disease-related pathways, including “arrhythmogenic right ventricular cardiomyopathy (ARVC),” “hypertrophic cardiomyopathy (HCM),” and “rheumatoid arthritis (RA),” were also significantly enriched. These pathways are closely related to intercellular signaling, immune regulation, and muscle structural integrity, indicating that CH2 deletion may disrupt transcriptional programs controlling ion transport, cytoskeletal dynamics, and stress adaptation (Figure 4B). These results highlight the potential role of CH2 in the regulation of cardiovascular and autoimmune disease–related pathways.

To evaluate the physiological impact of CH2 knockout, functional experiments were conducted. CCK-8 assays revealed a significant reduction in proliferation starting at 48 h (Figure 4C). To directly confirm the proliferation defect, we performed an EdU incorporation assay. Consistent with the CCK-8 results, CH2-KO cells exhibited a significantly lower rate of EdU incorporation, indicating a direct impairment in DNA synthesis and cell cycle progression (Figures 4D,E). While flow cytometry demonstrated increased late apoptosis in CH2-deficient cells (Figures 4F,G), indicating an essential role of CH2 in cell survival.

### 3.5 CH2 deletion triggers RA and HCM transcriptomic signatures

**FIGURE 5:**
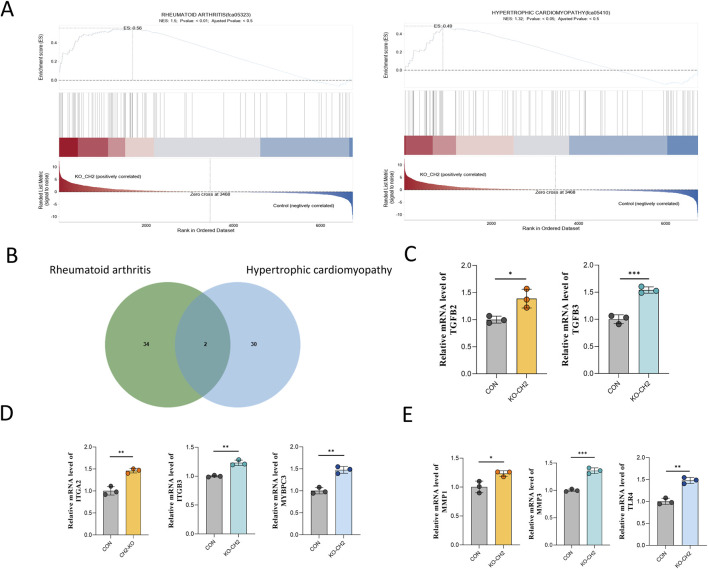
Transcriptomic convergence of RA and HCM pathways in CH2-KO cells. **(A)** Genome-wide expression dynamics and PCA of RNA-seq data from rheumatoid arthritis (left) and hypertrophic cardiomyopathy (right) models. The upper plots show expression trend curves, middle heatmaps display sample clustering, and lower plots present PCA distribution, indicating transcriptional remodeling and sample separation between disease and control groups. **(B)** Venn diagram illustrating the overlap of differentially expressed genes in models of rheumatoid arthritis (green circles) and hypertrophic cardiomyopathy (blue circles). Numbers indicate unique and shared genes. **(C)** Relative mRNA expression of *TGFB2* and *TGFB3*. **(D)** Relative mRNA expression of *ITGA2*, *ITGB3* and *MYBPC3*. **(E)** Relative mRNA expression of *MMP1*, *MMP3* and *TLR4*. Data were obtained from three independent experiments. Data are presented as mean ± standard deviation, and P values were calculated by two-tailed unpaired t-test. Significant differences are indicated by * (P < 0.05), ** (P < 0.001) and *** (P < 0.001).

To further delineate the molecular consequences of CH2 deletion, we focused on two significantly enriched disease gene sets: rheumatoid arthritis (RA) and hypertrophic cardiomyopathy (HCM), which were identified in our RNA-seq dataset. Gene Set Enrichment Analysis (GSEA) revealed that both RA- and HCM-associated gene sets were significantly enriched in CH2-KO samples compared to controls. Enrichment plots demonstrated that disease-related genes were predominantly clustered at the top of the ranked gene list, indicating coordinated upregulation and functional relevance (Figure 5A).

To identify specific disease-related genes contributing to this enrichment, Venn diagram analysis was performed, revealing 34 RA-specific genes, 30 HCM-specific genes, and two shared genes—*TGFB2* and *TGFB3*—both of which are known upstream regulators of inflammation and cardiac remodeling (Figure 5B).

We next selected representative genes for qPCR validation based on two criteria: (1) their established roles in RA or HCM pathology, and (2) consistent expression patterns in our transcriptomic data that mirrored known disease profiles. Notably, *ITGA2*, *ITGB3*, and *MYBPC3*, which are critical for cell adhesion and cardiac muscle architecture, were significantly upregulated (Figure 5D). The shared upstream regulators *TGFB2* and *TGFB3* also showed robust transcriptional activation (Figure 5C). In addition, innate immunity and ECM-related genes—including *MMP1*, *MMP3*, and *TLR4*—were markedly elevated (Figure 5E).

Collectively, these results suggest that CH2 deletion induces transcriptional reprogramming that activates convergent pathophysiological pathways associated with RA and HCM. The co-upregulation of *TGFB2* and *TGFB3* further supports their central roles as mediators linking inflammatory and structural cardiac remodeling processes, offering novel insights into potential mechanistic crosstalk and therapeutic intervention points.

## 4 Discussion

While targeted knockout of the CH2 gene using CRISPR/Cas9 offers a promising strategy to eliminate the major cat allergen Fel d1 and advance the development of hypoallergenic cats, our findings reveal that this genetic ablation induces extensive transcriptomic reprogramming in feline cells. Notably, we observed significant enrichment of signaling pathways associated with hypertrophic cardiomyopathy (HCM) and rheumatoid arthritis (RA), highlighting potential unintended biological risks that warrant thorough investigation.

In this study, we used CRISPR/Cas9 to specifically knock out the CH2 domain of Fel d1 and established a CH2-deficient cell line in primary feline cells, serving as potential nuclear donors for SCNT. Sequencing confirmed a specific 3-bp insertion (Figure 2B), introducing a frameshift mutation predicted to generate a premature termination codon. This is expected to produce a truncated, non-functional CH2 protein or trigger nonsense-mediated mRNA decay, consistent with the observed near-complete loss of CH2 transcript and Fel d1 protein (Figures 2C–E). To evaluate potential developmental abnormalities or disease risks in cloned offspring following CH2 deletion, we conducted systematic validation through gene editing, transcriptome sequencing, disease pathway enrichment, and functional cellular assays. The results revealed that CH2 knockout triggered extensive transcriptional reprogramming and significantly enriched pathways related to rheumatoid arthritis and hypertrophic cardiomyopathy, suggesting a potential disruption of cellular homeostasis. These findings provide theoretical support for the feasibility of generating hypoallergenic cats and emphasize the importance of functional assessment of gene-edited donor cells prior to SCNT. Furthermore, this study offers new insights into the potential roles and side effects of Fel d1 in feline physiology.

However, the current strategy faces limitations in editing efficiency. When using a single sgRNA construct in combination with Lipofectamine™ 3,000, and T7E1 analysis revealed editing efficiencies below 20%. This poses significant challenges for subsequent monoclonal isolation. These limitations are largely attributable to the inherent drawbacks of cationic lipid-based delivery systems, including low transfection efficiency (Wang et al., 2025), short expression windows (Masarwy et al., 2024), and strong cell-type dependency—issues widely reported in CRISPR delivery research (Kanduri et al., 2021). To address this, alternative delivery strategies may be considered. Viral vectors such as lentiviruses and adenoviruses have demonstrated transduction efficiencies exceeding 80% in epithelial models (Lyu et al., 2020), while advanced non-viral platforms like polyethyleneimine-functionalized PEG-b-PLGA nanoparticles can enhance endosomal escape and achieve up to 92% transfection efficiency in endothelial cells (Zhang et al., 2022). Furthermore, our current approach relies on a single-sgRNA design, which imposes additional limitations on editing outcomes. Studies in mice have shown that triple-sgRNA combinations can increase editing efficiency from 32% to 97.5% (Sunagawa et al., 2016), and similar trends have been observed in human TNBC cells and microglia (Syahrani et al., 2024). Thus, future efforts should explore the use of multiple sgRNAs alongside optimized delivery systems to achieve more robust and efficient Fel d1 gene knockout.

Although Fel d1-edited cats have been successfully produced via CRISPR/Cas9-mediated microinjection (Lee et al., 2024), there is currently no precedent for generating viable offspring through somatic cell nuclear transfer (SCNT) using gene-edited donor cells. As such, the potential disease risk introduced by donor cell editing remains uncertain. Studies have demonstrated that the CRISPR/Cas9 procedure can introduce unforeseen complex genomic alterations (Bi et al., 2025), and induce cellular senescence and inflammatory perturbations (Conti et al., 2025) in various cell types. If present in donor cells, these editing-induced aberrations are poised to synergistically disrupt epigenetic reprogramming in SCNT embryos, thereby compromising developmental potential and presenting long-term safety risks.

While previous studies have suggested that Fel d1 may be non-essential across feline species due to its homology with other non-critical proteins (Cleveland et al., 2024), the transcriptomic consequences of its knockout remain uncharacterized. To address this gap, we performed comprehensive RNA-seq analysis on CH2-knockout cells to assess gene expression changes and identify possible risks associated with altered transcriptional profiles. This provides foundational insight for the safe application of SCNT and furthers our understanding of Fel d1’s cellular role.

Transcriptome sequencing revealed that CH2 knockout induced marked transcriptional reprogramming, identifying 3,469 DEGs, including 2,410 upregulated and 1,059 downregulated genes. PCA demonstrated clear separation between the CH2-KO and control groups, indicating global dysregulation of intracellular signaling. GO enrichment analysis showed that DEGs were mainly enriched in calcium ion binding, transmembrane receptor activity, and extracellular matrix (ECM) organization—suggesting that CH2 deletion may impair ionic homeostasis, cell communication, and structural integrity. KEGG analysis further highlighted significant enrichment in disease pathways, particularly RA and HCM. Both RA and HCM are clinically significant in domestic cats (Hanna, 2005; Liu et al., 2023), with RA involving cytokine imbalance, innate immune activation and ECM degradation (Ainsworth et al., 2022), and HCM characterized by cardiomyocyte hypertrophy, adhesion dysfunction and calcium dysregulation (Santini et al., 2020; De Jong et al., 2022; Liu et al., 2023). The GO terms identified—calcium binding, transmembrane signaling, and ECM structure—are mechanistically consistent with these disease processes, supporting the biological relevance of the observed transcriptional changes. These findings provide foundational insights for the safe application of SCNT and advance our understanding of Fel d1’s cellular role.

To further investigate the potential disease risks associated with CH2 deletion, we performed KEGG-based gene set enrichment analysis (GSEA), We selected eight representative genes from these two disease modules for qPCR validation, confirming the transcriptomic trends. In the context of HCM, the upregulated genes were primarily involved in TGF-β family members (*TGFB2*, *TGFB3*), integrin (*ITGA2*, *ITGB3*), and *MYBPC3*. *MYBPC3* is one of the most common genetic determinants of feline HCM particularly in breeds such as Maine Coon and Ragdoll cats (Ali et al., 2025),. Mutations in *MYBPC3* have been directly linked to disease onset (Fabiani et al., 2025). *TGFB2* plays a pivotal role in cardiovascular remodeling by modulating smooth muscle cell (SMC) differentiation (Liu et al., 2019; Tang et al., 2025), *TGFB3* variants similarly contribute to HCM-like phenotypes (Perik et al., 2023). *ITGA2* is transcriptionally regulated by LncRNA-ITGA2 via enhancer–promoter interactions mediated by the DNA-binding protein NONO (Guo et al., 2025).

For RA, the enriched genes were primarily associated with TGF-β signaling (*TGFB2, TGFB3*), matrix metalloproteinases (*MMP1, MMP3*), and Toll-like receptor 4 (*TLR4*). TGF-β members are implicated in joint destruction and inflammation by regulating osteoclastogenesis and immune responses (Zeng et al., 2021; Xia et al., 2022). MMP3, a well-established mediator in RA pathology, promotes synovial hyperplasia and degrades the extracellular matrix, thereby accelerating cartilage erosion (Guo et al., 2024). TLR4 activation and its internalization through the Rab5a-dependent endosomal pathway enhance macrophage inflammatory responses, including the secretion of IL-6 and TNF-α (Chen et al., 2024). Furthermore, TLR4 also drives activation of the NLRP3 inflammasome, which is transcriptionally regulated by microRNAs such as miR-623 in synovial macrophages from RA patients (Yang et al., 2021; Yin et al., 2022). Together, these molecules play established roles in inflammatory remodeling and epithelial barrier responses (Cho et al., 2014; Zhang S. Z. et al., 2019). Therefore, our data point to the possibility that Fel d1 has a dual function, acting not only as an allergen but also as an immunomodulatory molecule. The transcriptomic shifts are consistent with the hypothesis that although Fel d1 deletion holds promise for reducing allergenicity, its absence could potentially perturb feline immune homeostasis, influencing key processes like allergen processing, cytokine release, or extracellular matrix regulation.

Together, these results demonstrate that CH2 deletion disrupts the expression of disease-associated genes central to fibrotic, inflammatory, and immune pathways. The concordance between GSEA-identified gene sets and qPCR data further underscores that CH2 deficiency may heighten susceptibility to HCM and RA-like phenotypes via transcriptomic reprogramming. Although CH2 knockout effectively reduces Fel d1, the unintended activation of RA- and HCM-related pathways highlights a potential trade-off between allergen reduction and cellular homeostasis. These findings emphasize the need for balanced risk assessment in developing hypoallergenic cats.

## 5 Conclusions

In summary, targeted deletion of the Fel d1 CH2 domain resulted in extensive transcriptional reprogramming in feline cells, with significant upregulation of disease-associated genes linked to HCM and RA. These alterations encompassed key regulators of TGF-β signaling, cell adhesion, innate immune responses, and extracellular matrix remodeling. Additionally, we observed abnormal expression of transcripts involved in intracellular signaling cascades and pro-inflammatory mediators, suggesting that CH2 deletion may broadly disrupt cellular homeostasis beyond specific disease pathways. While eight genes were validated, many other differentially expressed transcripts, including those related to signal transduction fidelity and cytokine regulation, remain unexplored. These unresolved transcriptional changes warrant further investigation to fully assess the systemic impact of Fel d1 editing. Comprehensive screening and functional profiling of donor cells will be essential to mitigate disease risk and enhance the safety and developmental success of SCNT-derived offspring.

## Data Availability

The datasets presented in this study can be found in online repositories. The names of the repository/repositories and accession number(s) can be found in the article/supplementary material.

## Abbreviations

Fel d1, Felis Domesticus Allergen 1; KO, knockout; CH2 gene, Fel d1 Chain 2; sgRNA, Single Guide RNA; PAM, Sprotospacer Adjacent Motifs; T7EI, T7 Endonuclease I; RNA-seq, RNA sequencing; DEGs, Differential Expressed Genes; PCA, principal component analysis; GO,Gene Ontology; KEGG, Kyoto Encyclopedia of Genes and Genomes; GSEA, Gene Set Enrichment Analysis; HCM, Hypertrophic Cardiomyopathy; RA, Rheumatoid Arthritis; SCNT, Somatic Cell Nuclear Transfer; DSBs, double-strand breaks.
